# Cluster analysis-based clinical phenotypes of idiopathic interstitial pneumonias: associations with acute exacerbation and overall survival

**DOI:** 10.1186/s12890-021-01428-3

**Published:** 2021-02-22

**Authors:** Yoichiro Aoshima, Masato Karayama, Yasuoki Horiike, Kazutaka Mori, Hideki Yasui, Hironao Hozumi, Yuzo Suzuki, Kazuki Furuhashi, Tomoyuki Fujisawa, Noriyuki Enomoto, Yutaro Nakamura, Naoki Inui, Takafumi Suda

**Affiliations:** 1 Second Division, Department of Internal Medicine, Hamamatsu University School of Medicine, 1-20-1 Handayama, Hamamatsu, 431-3192 Japan; 2 Department of Respiratory Medicine, Shizuoka City Shimizu Hospital, 1231 Miyakami, Shizuoka, 424-8636 Japan; 3 Department of Clinical Pharmacology and Therapeutics, Hamamatsu University School of Medicine, 1-20-1 Handayama, Hamamatsu, 431-3192 Japan

**Keywords:** Idiopathic pulmonary fibrosis, Interstitial lung disease, Interstitial pneumonia, IPAF, IPF

## Abstract

**Background:**

The precise classification of idiopathic interstitial pneumonia (IIP) is essential for selecting treatment as well as estimating clinical outcomes; however, this is sometimes difficult in clinical practice. Therefore, cluster analysis was used to identify the clinical phenotypes of IIPs, and its usefulness for predicting clinical outcomes was evaluated.

**Methods:**

Cluster analysis was performed using clinical features including patients’ demographics; histories; pulmonary function test data; and laboratory, physical and radiological findings.

**Results:**

In 337 patients with IIPs, four clusters were identified: Cluster I, in which > 80% of the patients had autoimmune features; Cluster II, which had the lowest rate of smoking, the lowest percent predicted forced vital capacity (%FVC) and the lowest body mass index (BMI); Cluster III, which had the highest rate of smoking, the highest rate of dust exposure, the second lowest %FVC and normal BMI; and Cluster IV, which exhibited maintenance of %FVC and normal BMI. Cluster IV had significantly longer overall survival than Clusters II and III. Clusters I and III had significantly longer overall survival than Cluster II. Clusters II and III had a significantly higher cumulative incidence of acute exacerbation than Cluster IV.

**Conclusion:**

Cluster analysis using clinical features identified four clinical phenotypes of IIPs, which may be useful for predicting the risk of acute exacerbation and overall survival.

## Background

Idiopathic interstitial pneumonias (IIPs) consist of heterogeneous interstitial lung diseases of unknown aetiology. Based on their clinical, radiological and pathologic features, IIPs comprise several disease entities, such as idiopathic pulmonary fibrosis (IPF), nonspecific interstitial pneumonia (NSIP) and cryptogenic organizing pneumonia (COP) [1].

The precise classification of IIPs is essential for selecting treatment and predicting prognosis; however, this is not simple in clinical practice [1]. First, diagnosis at a single point is sometimes difficult, but this can be achieved by monitoring disease behaviour. For example, some patients with IIPs lacking honeycombing experience disease progression over several years, and they develop a usual interstitial pneumonia (UIP) pattern characterized by honeycombing on high-resolution computed tomography (HRCT) that is eventually diagnosed as IPF. In these patients, it is difficult to accurately diagnose the disease in the first examination. Additionally, patients with advanced IIPs other than IPF sometimes develop radiological and pathological honeycombing that mimics IPF [2]. Second, pathological patterns can vary among different sites of the lungs in patients with IIPs [3]. If surgical lung biopsy was not performed for representative lesions, it is difficult to ensure an accurate diagnosis [4, 5]. Furthermore, discordance in diagnosis even among specialists is frequent [6, 7]. Third, surgical lung biopsy, an essential procedure for the diagnosis of IIPs, cannot be performed in all patients [8]. In fact, according to a recent ATS/ERS/JRS/ALAT guideline, surgical lung biopsy is not necessarily required for the diagnosis of IPF in patients with typical radiologic findings of UIP [9]. Transbronchial cryobiopsy has attracted attention as a less invasive procedure than surgical lung biopsy; however, its utility for the diagnosis of IIPs remains a matter of debate [10, 11].

A new trend of classifying interstitial lung diseases (ILDs) using clinical characteristics, regardless of conventional disease entities, has emerged [12]. Recently, the concept of progressive fibrosing ILD (PF-ILD) was proposed, and this concept covers several IIPs featuring self-sustaining fibrosis, a progressive decline in lung function and early mortality [12]. PF-ILD comprises a comprehensive group of ILDs, including IIPs, connective tissue disease-associated ILD (CTD-ILD), sarcoidosis and chronic hypersensitivity pneumonia (CHP). Although the concept of PF-ILD has not been validated, it is expected to play a certain role from a therapeutic perspective [13]. Interstitial pneumonia with autoimmune features (IPAF) is another attempt to identify IIPs with features suggestive of, but not definitive for, CTD-ILD [14]. The diagnostic criteria of IPAF are positivity for two of the following three domains: clinical domain (extra-thoracic symptoms associated with autoimmune diseases), serologic domain (serum autoantibodies) and morphologic domain (HRCT and histopathologic findings and multi-compartment involvement other than IIPs) [14].

Clinical phenotyping using cluster analysis, which groups subjects according to the similarities and differences of their clinical features, has recently attracted attention for classifying heterogeneous diseases. For example, clinical phenotypes determined via cluster analysis using clinical data reveal distinct clinical outcomes in asthma and COPD [15, 16]. In all ILDs, clinical phenotypes determined using cluster analysis illustrated considerable predictive accuracy for clinical outcomes [17]. However, more than one-third of studied patients had non-IIPs such as CTD-ILD and CHP, both of which exhibit distinct characteristics and different clinical outcomes from IIPs [17].

We hypothesized that the clinical phenotypes of IIPs provide more useful information about clinical outcomes among patients. In this study, we performed cluster analysis using the clinical data of patients with IIPs and evaluated the clinical utility of the phenotypes.

## Methods

### Study design

This retrospective observational study followed the ethical standards of the Declaration of Helsinki. The study protocol was approved by the Institutional Review Board of Hamamatsu University School of Medicine (Hamamatsu, Japan, approval No. E15-197).

### Patients

The medical records of consecutive patients with IIPs who were diagnosed at Hamamatsu Medical University Hospital between September 2004 and August 2018 were retrospectively analysed. The IIP diagnosis followed American Thoracic Society (ATS)/European Respiratory Society (ERS) guidelines [1, 9, 18, 19]. Patients who exhibited acute exacerbation (AE) at the first visit were excluded [20].

### Data collection

The following data were collected at the time of IIP diagnosis: age, sex, body mass index (BMI), pack-year smoking history, history of dust exposure, laboratory data (C-reactive protein [CRP], lactate dehydrogenase [LDH], albumin, Krebs von den Lungen-6 [KL-6], surfactant protein D [SP-D]), pulmonary function (percent predicted forced vital capacity [%FVC], percent predicted forced expiratory volume in 1 s [%FEV_1_]) and the clinical and pathological diagnoses (if performed). The history of dust exposure was based on each patient’s reported history. Dust was classified as organic or inorganic, and the type of exposure was classified as occupational or environmental. The existence of emphysema and honeycombing was evaluated via chest high-resolution computed tomography (HRCT) at the diagnosis. The definitions of emphysema and honeycombing were described elsewhere [21]. Chest HRCT data were reviewed by two independent observers who were masked to patient data. Autoimmune features were recorded according to the IPAF diagnostic criteria [14].

### Statistical analysis

Hierarchical clustering was performed using age, sex, BMI, histories of smoking and dust exposure, autoimmune features, laboratory data (CRP, LDH, albumin, KL-6, SP-D), pulmonary function (%FVC, %FEV_1_) and HRCT findings (emphysema, honeycombing) to identify clinical IIP subtypes. The number of clusters in which the scree plot of the distances between the clusters in a dendrogram of hierarchical clustering rose sharply, indicating different characteristics between clusters, was determined. Overall survival (OS) and the time to the first acute exacerbation (AE) were measured from the IIP diagnosis. The Kaplan–Meier method and log-rank test were used to analyse OS, and Grey’s test was used to analyse the time to the first AE. Wilcoxon’s signed-rank test was used for continuous variables, and Fisher’s exact test was applied for categorical variables. All comparisons among clusters were adjusted using Bonferroni’s correction. Data were expressed as the median (range) unless otherwise indicated. All statistical tests were two-sided, and *p* < 0.05 indicated significance. All values were analysed using EZR (Saitama Medical Center, Jichi Medical University, Saitama, Japan), which is a graphical user interface for R (The R Foundation for Statistical Computing, Vienna, Austria), except the cluster analysis, which was performed using JMP v13.0.0 (SAS Institute Japan, Tokyo, Japan).

## Results

### Baseline characteristics

**Fig. 1:**
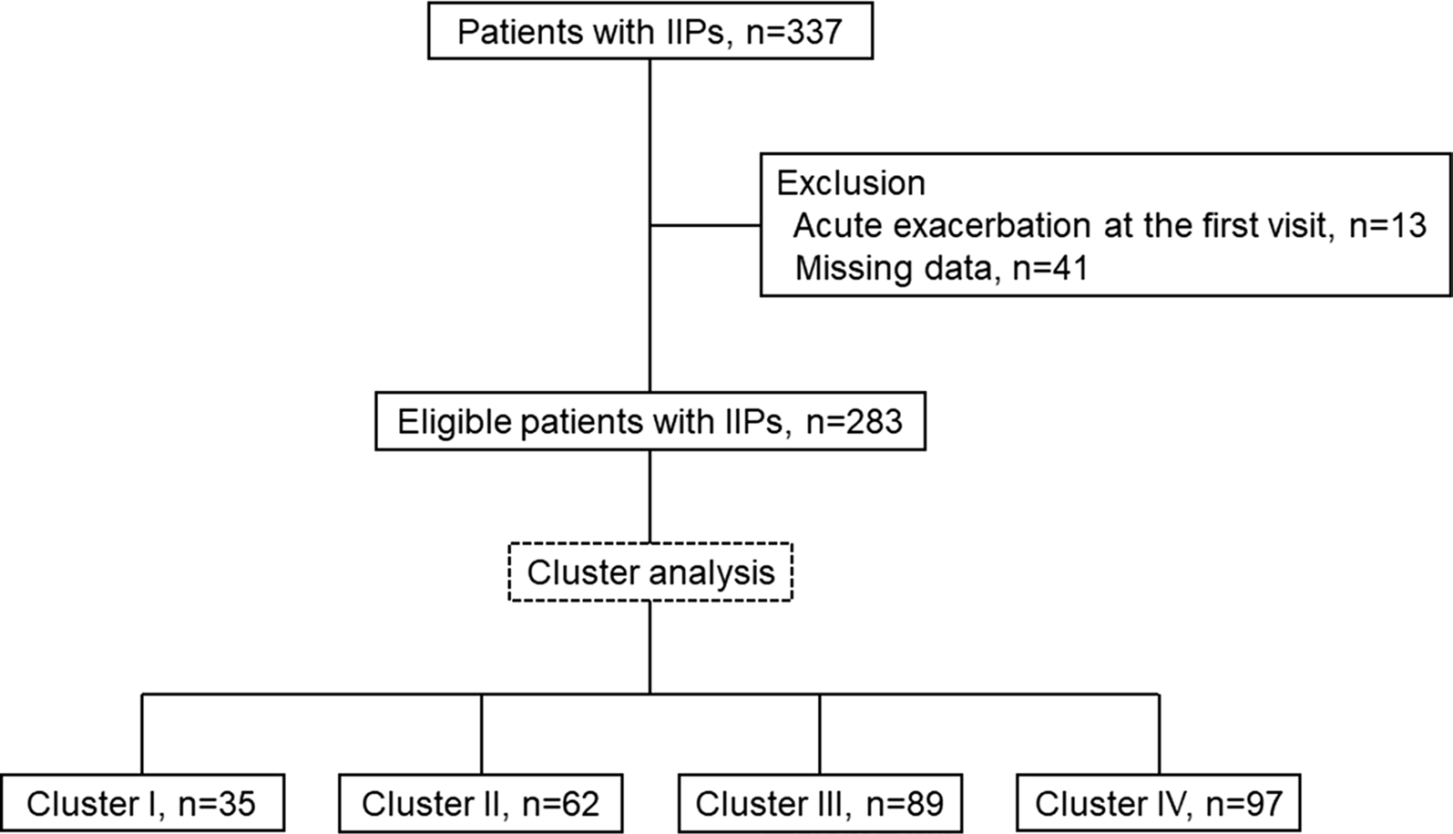
Flow diagram of the study patients. IIP, idiopathic interstitial pneumonia

In total, 337 patients with IIPs were screened, and 54 were excluded because of missing data (n = 41) and AE at the time of diagnosis (n = 13), leaving 283 patients (Fig. 1). The clinical characteristics of these patients are presented in Table 1. The study cohort consisted mainly of males, and more than half of the patients had smoking histories. In total, 148 (52.2%) patients had %FVC < 80%, and 134 (47.3%) patients had %FEV_1_ < 80%. Among 69 (24.3%) patients with histories of dust exposure, organic and inorganic materials were responsible for 2.9 and 97.1% of cases, respectively, and all instances of exposure were associated with occupational exposure. Sixty-eight (24.0%) patients underwent surgical lung biopsy. Additionally, 94 (33.2%), 19 (6.7%), 16 (5.6%), 12 (4.2%) and 4 (1.4%) patients were diagnosed with IPF (clinical, n = 66; pathological, n = 28), COP, NSIP, pleuroparenchymal fibroelastosis (PPFE) and desquamative interstitial pneumonia (DIP)/respiratory bronchiolitis-associated interstitial lung disease (RB-ILD), respectively (Table 2). The remaining 138 patients (48.7%) had unclassifiable IIPs. Thirty-nine (13.7%) patients had autoimmune features, including 6 (17.4%), 39 (100%) and 36 (92.3%) patients in the clinical, serologic and morphologic domains, respectively (Table 2). The median observation time was 39.4 months (range 1.0–165.0 months).

**Table 1:** Characteristics of the study patients

	All, n = 283	Cluster I, n = 35	Cluster II, n = 62	Cluster III, n = 89	Cluster IV, n = 97
Age, years	70.6 (20–90)	70.7 (52–83)^#^	75.9 (55–90)^$, ¶^	68.9 (20–89)	69.2 (41–88)
Sex, male	214 (75.6)	22 (62.8)^$^	49 (79.0)^$, ¶^	83 (93.2)^¶^	60 (61.8)
Body mass index, kg/m^2^	22.8 (13.1–36.8)	22.9 (18.1–29.7)^#^	20.8 (13.1–25.8)^$, ¶^	24.9 (19.4–36.8)^¶^	22.1 (14.9–29.8)
Smoking history	182 (64.3)	19 (54.2)^$^	31 (50.0)^$^	79 (88.7)^¶^	53 (54.6)
Pack-year smoking	42 (2.5–165)	30 (18.8–66)^$^	40 (5–100)^$^	49 (5–165)^¶^	40 (2.5–90)
Dust exposure	69 (24.3)	10 (28.5)^#^	6 (9.6)^$^	40 (44.9)^¶^	13 (13.4)
Organic / inorganic	2 (0.7)/ 67 (23.6)	0 (0) / 10 (28.5)	0 (0) / 6 (9.6)	1 (1.1) / 39 (43.8)	1 (1.0) / 12 (12.3)
Spirometry					
FVC, L	2.48 (0.57–4.85)	2.51 (0.91–4.16)^#^	1.82 (0.57–3.95)^$, ¶^	2.55 (1.41–3.95)	2.82 (0.97–4.85)
% predicted FVC	78.5 (27.6–124.0)	81.8 (31.6–113.0)^#^	64.5 (27.6–116.0)^$, ¶^	73.0 (39.8–112.0)^¶^	89.1 (40.9–124.0)
FEV_1_, L	1.98 (0.57–3.95)	1.97 (0.82–3.02)^#^	1.72 (0.57–3.95)^$, ¶^	2.02 (1.19–3.16)	2.31 (0.74–3.89)
% predicted FEV_1_	81.0 (31.9–131.6)	82.3 (66.1–114.0)^#, $^	68.9 (31.9–100.0)^$, ¶^	72.4 (41.4–117.0)^¶^	87.6 (61.0–131.6)
FEV_1_/FVC, %	82.4 (53.4–100.0)	81.7 (65.8–100.0)	87.5 (63.4–100.0) ^$^	81.0 (53.4–98.7)	82.3 (61.3–98.8)
Laboratory data					
CRP, mg/dL	0.20 (0.01–25.5)	0.27 (0.01–4.8)^#, ¶^	1.13 (0.02–25.5)^$, ¶^	0.23 (0.02–9.0)^¶^	0.10 (0.01–4.7)
LDH, U/L	225 (94–569)	230 (94–319)^¶^	213 (132–450)^$^	241 (170–569)^¶^	205 (141–334)
Albumin, g/dL	4.1 (1.9–4.9)	4.0 (2.4–4.9)^#, $, ¶^	3.6 (1.9–4.5)^$, ¶^	4.1(2.8–4.8)^¶^	4.2 (3.4–4.9)
KL-6, U/mL	829 (112–5710)	1010 (164–3651)	664 (112–2070)^$^	1148 (177–5710)^¶^	644 (196–3378)
SP-D, ng/mL	179 (17.2–1160)	166 (25.4–533)^$^	173 (33.4–1130)^$^	218 (49.8–1160)^¶^	154 (17.2–525)
CT findings					
Emphysema	90 (31.8)	11 (31.4)	14 (22.5)^$^	35 (39.3)	30 (30.9)
Honeycombing	82 (28.9)	5 (14.2)^#, $^	23 (37.0)^¶^	34 (38.2)^¶^	20 (20.6)
Treatments					
Steroids	96 (33.9)	15 (42.8)	21 (33.8)	36 (40.4)^¶^	24 (24.7)
Immunosuppressants	35 (12.3)	4 (11.4)	5 (8.0)	17 (19.1)	9 (9.2)
Antifibrotic agents	56 (19.7)	3 (8.5)^$^	13 (20.9)	28 (31.4)^¶^	12 (12.3)

Data are presented as the median (range) or number (%)

CRP, C-reactive protein; FEV_1_, forced expiratory volume in 1 s; FVC, forced vital capacity; IIPs, idiopathic interstitial pneumonias; IPAF, interstitial pneumonia with autoimmune features; KL-6, Krebs von den Lungen-6; LDH, lactate dehydrogenase; SP-D, pulmonary surfactant protein-D

^#^*p* < 0.05 compared with Cluster II

^$^*p* < 0.05 compared with Cluster III

^¶^*p* < 0.05 compared with Cluster IV

**Table 2:** Characteristics of the study patients

	All, n = 283	Cluster I, n = 35	Cluster II, n = 62	Cluster III, n = 89	Cluster IV, n = 97
IPF	94 (33.2)	6 (17.1)	22 (35.4)	46 (51.6)	20 (20.6)
NSIP	16 (5.6)	5 (14.2)	0 (0)	5 (5.6)	6 (6.1)
COP	19 (6.7)	3 (8.5)	8 (12.9)	3 (3.3)	5 (5.1)
DIP / RB-ILD	4 (1.4)	2 (5.7)	0 (0)	0 (0)	2 (2.0)
PPFE	12 (4.2)	0 (0)	8 (12.9)	0 (0)	4 (4.1)
Unclassifiable IIPs	138 (48.7)	19 (54.2)	24 (38.7)	35 (39.3)	60 (61.8)
Autoimmune features	39 (13.7)	30 (85.7)	6 (9.6)	1 (1.1)	2 (2.0)

Data are expressed as number (%)

COP, cryptogenic organizing pneumonia; DIP, desquamative interstitial pneumonia; IIP, idiopathic interstitial pneumonia; IPF, idiopathic pulmonary fibrosis; NSIP, non-specific interstitial pneumonia; PPFE, pleuroparenchymal fibroelastosis; RB-ILD, respiratory bronchiolitis-associated interstitial lung disease

### Clinical characteristics of the clusters

Four clusters were identified in the cluster analysis using clinical data (Additional file 1: Fig. 1).

**Fig. 2:**
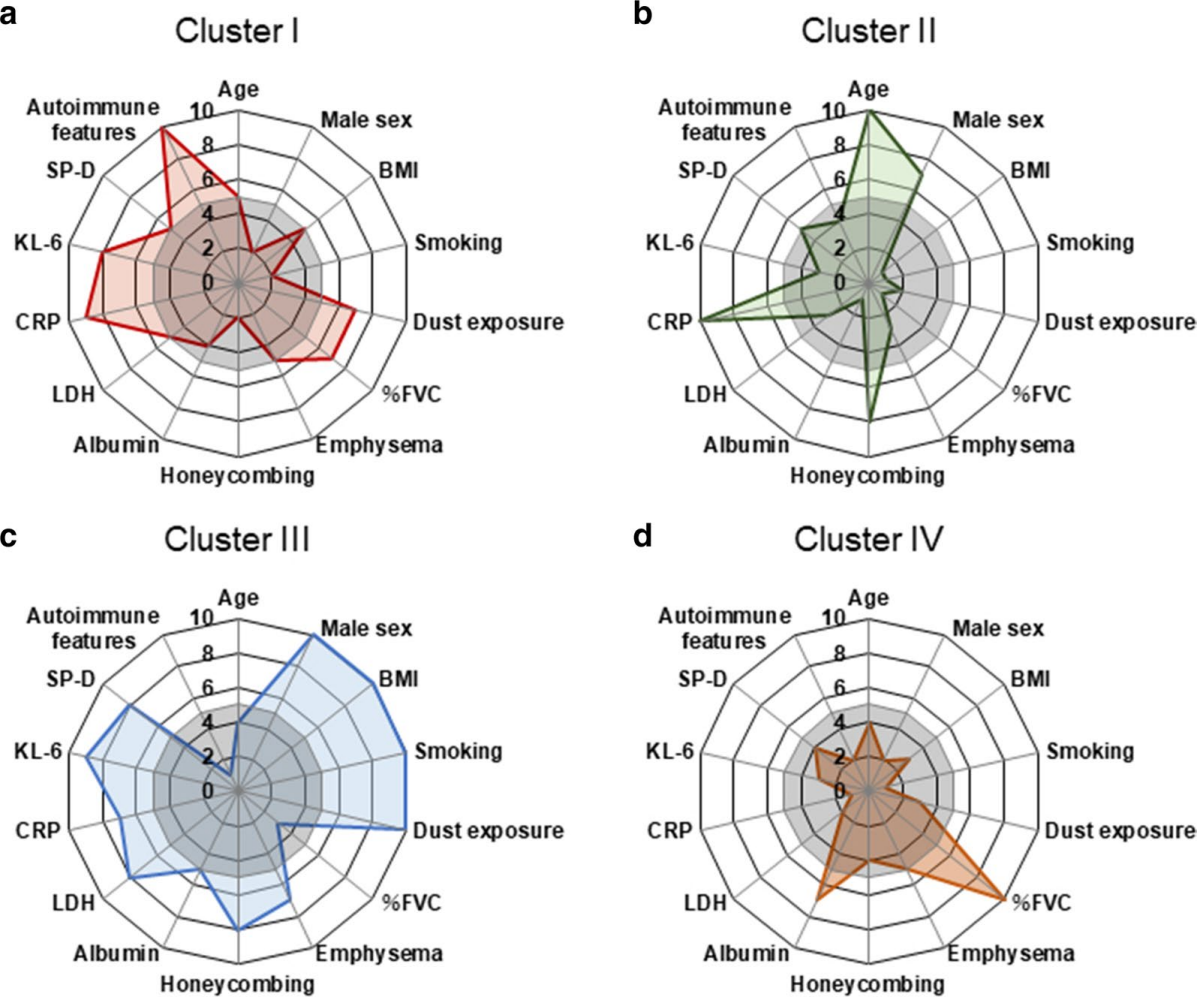
Radar plot of clinical features of the four clusters. **a** Cluster I, **b** Cluster II, **c** Cluster III, and **d** Cluster IV. Plot scale: 1, < 0.4 SDs below the mean; 2, 0.3–0.4 SDs below the mean; 3, 0.2–0.3 SDs below the mean; 4, 0.1–0.2 SDs below the mean; 5, mean to 0.1 SDs below the mean; 6, mean to 0.1 SDs above the mean; 7, 0.1–0.2 SDs above the mean; 8, 0.2–0.3 SDs above the mean; 9, 0.3–0.4 SDs above the mean; 10, > 0.4 SDs above the mean. BMI, body mass index; CRP, C-reactive protein; IPAF, interstitial pneumonia with autoimmune features; KL-6, Krebs von den Lungen-6; %FVC, percent predicted forced vital capacity; SP-D, pulmonary surfactant protein-D

Cluster I (n = 35) was an intermediate-aged cohort (70.7 years) with low proportions of males (62.8%) and smokers (54.2%; Table 1, Fig. 2a). Cluster I featured preserved %FVC (81.8%), the lowest honeycombing rate (14.2%) and the highest rate of autoimmune features (85.7%). Regarding laboratory data, Cluster I had the second highest KL-6 level (1010 U/mL), a slightly increased SP-D level (166 ng/mL) and normal CRP, LDH and albumin levels. In Cluster I, six (17.1%), five (14.2%), three (8.5%) and two (5.7%) patients were diagnosed with IPF (pathological, n = 3; clinical, n = 3), NSIP, COP and DIP, respectively. The remaining 19 (54.2%) patients had unclassifiable IIP (Table 2).

Cluster II (n = 62) had the oldest population (75.9 years), the smallest percentage of smokers (50.0%), the lowest BMI (20.8 kg/m^2^) and the lowest %FVC (64.5%; Table 1, Fig. 2b), as well as the second largest male population (79.0%). Emphysema was least frequent in this group (22.5%), but the second highest rate of honeycombing was noted (37.0%). In laboratory findings, Cluster II had the highest CRP level (1.13 mg/dL), the lowest serum albumin level (3.6 g/dL) and slightly increased KL-6 (664 U/mL) and SP-D levels (173 ng/mL). In total, 22 (35.4%), 8 (12.9%) and 8 (12.9%) patients were diagnosed with IPF (clinical, n = 20; pathological, n = 2), PPFE and COP, respectively. The remaining 24 (38.7%) patients had unclassifiable IIP (Table 2).

Cluster III (n = 89) had the youngest population (68.9 years), the highest proportion of males (93.2%), the highest BMI (24.9 kg/m^2^) and the highest frequencies of smoking (88.7%) and dust exposure (44.9%; Table 1, Fig. 2c). This cluster had the second lowest %FVC (73.0%). Cluster III had the highest LDH (241 U/L), KL-6 (1148 U/mL) and SP-D levels (218 ng/mL) and normal CRP and albumin levels. The rates of emphysema and honeycombing were highest in this group (39.3 and 38.2%, respectively). In total, 46 (51.6%), 5 (5.6%) and 3 (3.3%) patients were diagnosed with IPF (clinical, n = 29; pathological, n = 17), NSIP and COP, respectively. The remaining 35 (39.3%) patients had unclassifiable IIP (Table 2).

Cluster IV (n = 97) had the second youngest population (69.2 years), the highest proportion of females (38.2%), normal BMI (22.1 kg/m^2^) and lower frequencies of smoking (54.6%) and dust exposure (13.4%; Table 1, Fig. 2d). Cluster IV had the highest %FVC (89.1%) and normal CRP, LDH and albumin levels. Cluster IV had the lowest KL-6 (644 U/mL) and SP-D levels (154 ng/mL). The rates of emphysema and honeycombing were 30.9 and 20.6%, respectively. Overall, 20 (20.6%), 6 (6.1%), 5 (5.1%), 4 (4.1%) and 2 patients (2.0%) were diagnosed with IPF (clinical, n = 13; pathological, n = 7), NSIP, and COP, PPFE and DIP, respectively. The remaining 60 (61.8%) patients had unclassifiable IIPs (Table 2).

### Differences in clinical outcomes among the clusters

**Fig. 3:**
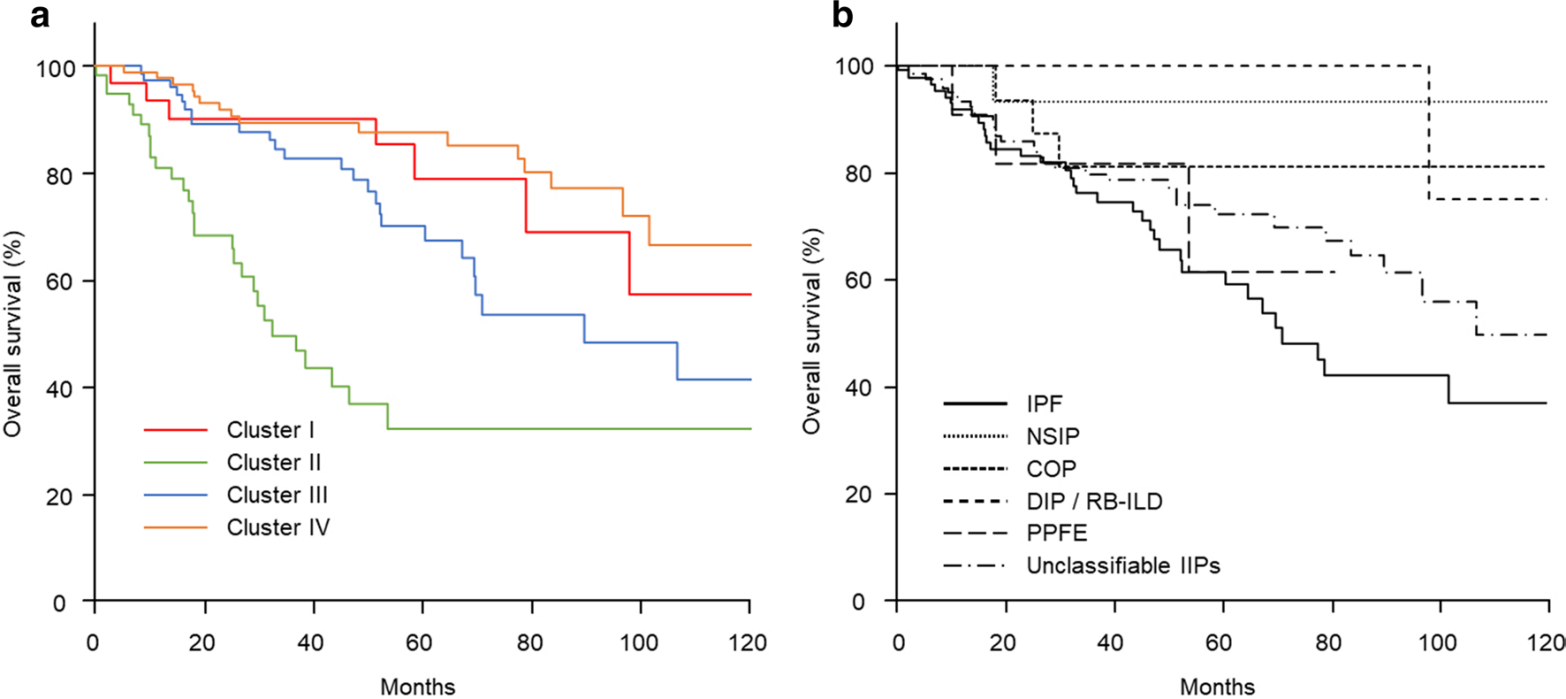
Overall survival (OS). **a** OS was significantly longer in Cluster IV (orange line) than in Clusters I (red line, *p* = 0.53), II (green line, *p* < 0.01), and III (blue line, *p* = 0.01). OS was significantly longer in Cluster I than in Clusters II (*p* < 0.01) and III (*p* = 0.21). OS was significantly longer in Cluster III than in Cluster II (*p* < 0.01). **b** Patients with idiopathic pulmonary fibrosis (IPF, continuous line) had significantly longer OS than those with non-specific interstitial pneumonia (NSIP, dotted line, *p* = 0.01), whereas there was no significant difference among the other idiopathic interstitial pneumonia groups. COP, cryptogenic organizing pneumonia (short dashed line); DIP/RB-ILD, desquamative interstitial pneumonia/respiratory bronchitis-associated interstitial lung disease (middle dashed line); PPFE, pleuroparenchymal fibroelastosis (long dashed line)

OS was significantly longer in Cluster IV than in Clusters II (*p* < 0.01) and III (*p* = 0.01; Fig. 3a). Clusters I and III had significantly longer OS than Cluster II (both *p* < 0.01). The 5-year OS rates were 87.7%, 79.0%, 70.1% and 32.3% in Clusters IV, I, III and II, respectively, and the 10-year OS rates were 66.6%, 57.6%, 41.4% and 32.3%, respectively.

**Fig. 4:**
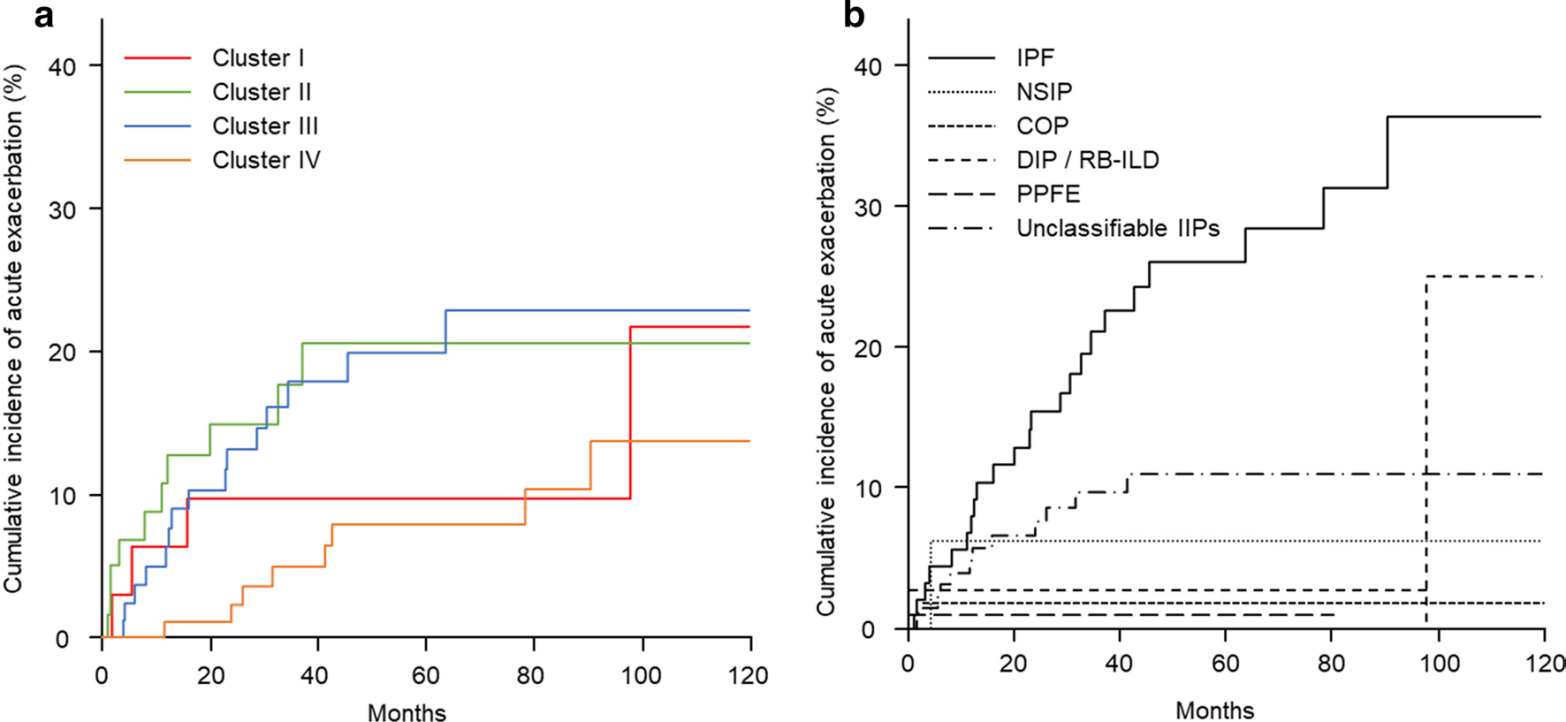
Cumulative incidence of acute exacerbation. **a** Clusters II (green line) and III (blue line) had significantly higher cumulative incidence rates of acute exacerbation than Cluster IV (orange line, both *p* = 0.03). **b** Patients with idiopathic pulmonary fibrosis (IPF, continuous line) had a significantly higher cumulative incidence rate of acute exacerbations than those with cryptogenic organizing pneumonia (COP, small dashed line, *p* = 0.01) and unclassifiable IIPs (long dashed short dashed line, *p* < 0.01), whereas there was no significant difference among the other idiopathic interstitial pneumonia groups. DIP/RB-ILD, desquamative interstitial pneumonia/respiratory bronchitis-associated interstitial lung disease (middle dashed line); NSIP, non-specific interstitial pneumonia (dotted line); PPFE, pleuroparenchymal fibroelastosis (long dashed line)

Clusters II and III had significantly higher cumulative incidences of AE than Cluster IV (both *p* = 0.03; Fig. 4a). The 5-year cumulative incidence rates of AE were 20.6%, 19.9%, 9.7% and 8.0% in Clusters II, III, I, and IV, respectively, and the 10-year cumulative incidence rates were 20.6%, 23.0%, 21.7% and 13.8%, respectively.

Next, OS and AE were evaluated according to the IIP diagnosis. Patients with NSIP had significantly longer OS than those with IPF (*p* = 0.01), whereas there was no significant difference among the other groups (Fig. 3b). Patients with IPF had a significantly higher cumulative incidence of AE than those with COP (*p* = 0.01) and unclassifiable IIPs (*p* < 0.01), whereas there was no significant difference among the other groups (Fig. 4b).

### Alternative clustering analysis

If we had employed three clusters, Clusters III and IV would have been merged (Additional file 1: Fig. 1). Alternatively, if we had employed five clusters, Cluster II would have been divided into two clusters. One of the new clusters had a significantly lower proportion of males, lower BMI, lower %FVC, lower %FEV_1_, lower CRP, lower LDH and higher albumin than the other cluster (Additional file 2: Tables 4–5) However, OS and the cumulative incidence of AE did not differ between the clusters (Additional file 1: Figs. 2 and 3).

## Discussion

Four IIP clusters with distinct clinical features and outcomes were identified. Cluster I, in which > 80% of the patients had autoimmune features, featured a low risk for AE and good OS. Clusters II and III had the greatest decreases in %FVC and the highest honeycombing rates. More than 80% of patients with IPF belonged to these two clusters. Cluster II had the lowest %FVC, BMI and albumin levels. Cluster III had the second lowest %FVC and normal BMI and albumin levels, as well as the highest LDH, KL-6 and SP-D levels. OS was worst in Cluster II, followed by Cluster III. Both clusters had high risks of AEs. Cluster IV, representing the mildest IIPs with preserved %FVC and almost normal laboratory data, had the best OS and a low risk of AE. In our patients, more than half did not undergo surgical lung biopsies. Additionally, the radiologic findings used in the cluster analysis were simple; namely the existence of honeycombing and emphysema on chest CT. Our clustering of patients with IIPs using clinical data is highly feasible in practice, and this strategy may provide useful information about patients’ clinical outcomes, even without detailed pathological or radiologic findings.

Interestingly, our cluster analysis revealed that the clinical features associated with collagen vascular diseases were important components of the clinical phenotypes of IIPs. A considerable number of patients with IIPs have some features of collagen vascular disease without fulfilling the defined criteria of any collagen vascular diseases. Several classifications have been proposed for such patients, but controversies remain unresolved [22–25]. The IPAF concept was proposed by ATS/ERS to establish a unified platform for such patients with IIPs [14]. Several studies reported that patients with IPAF have better OS and lower risks of AE than their counterparts [26, 27]. Similarly, Cluster I, in which most patients had autoimmune features, was linked to better clinical outcomes than Clusters II and III. Additionally, patients with IPAF are younger and more commonly female than those without IPAF, coinciding with the characteristics of Cluster I [28–30]. The clinical significance and utility of the IPAF concept are poorly validated. Additionally, several studies reported that the IPAF criteria must be revisited. In this context, our simple clustering successfully extracts patients with IIPs and autoimmune features who have distinct outcomes (Cluster I).

Clusters II and III were characterized by advanced fibrosis accompanied by decreased %FVC and honeycombing, which may lead to poor prognoses and high risks of AE. Most study patients with IPF belonged to these clusters. Patients with IPF have higher AE rates and worse OS than those with other IIPs [31]. The high prevalence of IPF might have strong effects on clinical outcomes in Clusters II and III. However, other factors determined the characteristics of these clusters. More than 50% of patients in these clusters had non-IPF IIPs. Specifically, OS was poorest in Cluster II despite its lower proportion of patients with IPF than Cluster III. In this study, we identified age, low serum albumin levels, decreased %FVC and radiologic honeycombing as independent risk factors for OS in all patients with IIP (Additional file 2: Table 1). Additionally, the latter two were also risk factors for AE (Additional file 2: Table 2). Among the two clusters, Cluster II was associated with significantly older patient age, lower serum albumin levels, lower %FVC and higher rates of radiologic honeycombing than Cluster III. These differences may explain the clinical outcomes of Clusters II and III.

A previous study reported the usefulness of four clinical clusters for predicting clinical outcomes in 770 patients with ILDs [17]. The study included non-IIPs including CTD-ILD and CHP and diverse ethnicities (mainly Caucasian and secondarily African American). Therefore, the clusters were not completely consistent with those in the current study. However, the cluster with female-dominant demographics, elevated antinuclear antibody levels, and the best clinical outcomes and another cluster of elderly male smokers with coexisting emphysema and the second worst outcomes were similar to Clusters I and III, respectively, in the current study.

The current study had several limitations. First, the numbers of clusters affected the results. In the current study, we also considered the use of three or five clusters. If we had employed three clusters, the distinct clinical features of Clusters III and IV would have been lost. Meanwhile, if we had employed five clusters, the two new clusters derived from Cluster II would have included small numbers of patients (insufficient for reaching statistical significance); thus, they were grouped together. Second, the numbers and/or optimal combination of variables used for the cluster analysis were not validated. We employed clinical variables that could be representative of demographic, historical, physical, laboratory and radiographic information. However, there were limited data available because the study was retrospective. However, there were limited data available because the study was retrospective. For example, a considerable number of study patients lacked data for diffusion capacity of the lung for carbon monoxide, which would have provided important information regarding the outcomes of ILD. The selection of variables can vary according to the purpose of the classification, such as predicting OS, AE risk, therapeutic response. Further studies are warranted to optimize the clinical classifications of IIPs.

## Conclusion

Cluster analysis using simple clinical data identified four phenotypes from the heterogeneous group of IIPs. Even without surgical lung biopsy, the four clinical phenotypes were linked to distinct differences in AE risks and OS, which may help to predict clinical outcomes and make treatment decisions.

## Supplementary Information

**Additional file 1.** **Supplementary Figures 1–3: Figure 1**. Dendrogram of patients with IIPs using cluster analysis. **Figure 2**. Overall survival in the new clusters derived from Cluster II. **Figure 3**. Cumulative incidence of acute exacerbations in the new clusters derived from Cluster II.

**Additional file 2. Supplementary Tables 1–4: Table 1**. Cox proportional hazard analysis of overall survival. **Table 2**. Cox proportional hazard analysis of acute exacerbations.  **Table 3**. Characteristics of the new clusters derived from Cluster II. **Table 4**. Diagnosis of IIPs in the new clusters derived from Cluster II.

## Data Availability

The datasets used and/or analyzed during the current study are available from the corresponding author on reasonable request.

## Abbreviations

ATS: American Thoracic Society
BMI: Body mass index
CHP: Chronic hypersensitivity pneumonia
COP: Cryptogenic organizing pneumonia
COPD: Chronic obstructive pulmonary disease
CPFE: Combined pulmonary fibrosis and emphysema
CRP: C-reactive protein
CTD-ILD: Connective tissue disease-associated interstitial lung diseases
DIP: Desquamative interstitial pneumonia
ERS: European Respiratory Society
FEV_1_: Forced expiratory volume in 1 s
FVC: Forced vital capacity
HRCT: High resolution computed tomography
KL-6: Krebs von den Lungen-6
IIP: Idiopathic interstitial pneumonia
ILD: Interstitial lung disease
IPAF: Interstitial pneumonia with autoimmune features
IPF: Idiopathic pulmonary fibrosis
LDH: Lactate dehydrogenase
NSIP: Nonspecific interstitial pneumonia
OS: Overall survival
PPFE: Pleuroparenchymal fibroelastosis
SP-D: Surfactant protein D
%FEV_1_: Percent predicted forced expiratory volume in 1 s
%FVC: Percent predicted forced vital capacity
