# Supplementary material for: Cluster analysis-based clinical phenotypes of idiopathic interstitial pneumonias: associations with acute exacerbation and overall survival
Source: BMC Pulm Med. 2021 Feb 22;21:63. doi: 10.1186/s12890-021-01428-3 (PMC7898746; doi:10.1186/s12890-021-01428-3)
Supplement: Supplementary file 1 — Additional file 1. Supplementary Figures 1–3: Figure 1. Dendrogram of patients with IIPs using cluster analysis. Figure 2. Overall survival in the new clusters derived from Cluster II. Figure 3. Cumulative incidence of acute exacerbations in the new clusters derived from Cluster II. [file 12890_2021_1428_MOESM1_ESM.docx]

**Cluster analysis-based clinical phenotypes of idiopathic interstitial pneumonias: Associations with acute exacerbation and overall survival**

**Authors:** Yoichiro Aoshima, MD^1^, Masato Karayama, MD, PhD^1^, Yasuoki Horiike, MD^1^, Kazutaka Mori, MD, PhD^2^, Hideki Yasui, MD, PhD^1^, Hironao Hozumi, MD, PhD^1^, Yuzo Suzuki, MD, PhD^1^, Kazuki Furuhashi, MD, PhD^1^, Tomoyuki Fujisawa, MD, PhD^1^, Noriyuki Enomoto, MD, PhD^1^, Yutaro Nakamura, MD, PhD^1^, Naoki Inui, MD, PhD^1,3^, Takafumi Suda, MD, PhD^1^

**Affiliations:**

^1^Second Division, Department of Internal Medicine, Hamamatsu University School of Medicine, 1-20-1 Handayama, Hamamatsu 431-3192, Japan

^2^Department of Respiratory Medicine, Shizuoka City Shimizu Hospital, 1231 Miyakami, Shizuoka, 424-8636, Japan

^3^Department of Clinical Pharmacology and Therapeutics, Hamamatsu University School of Medicine, 1-20-1 Handayama, Hamamatsu 431-3192, Japan

**Corresponding author:** Masato Karayama MD, PhD

**Supplementary Figure 1. Dendrogram of patients with IIPs using cluster analysis**


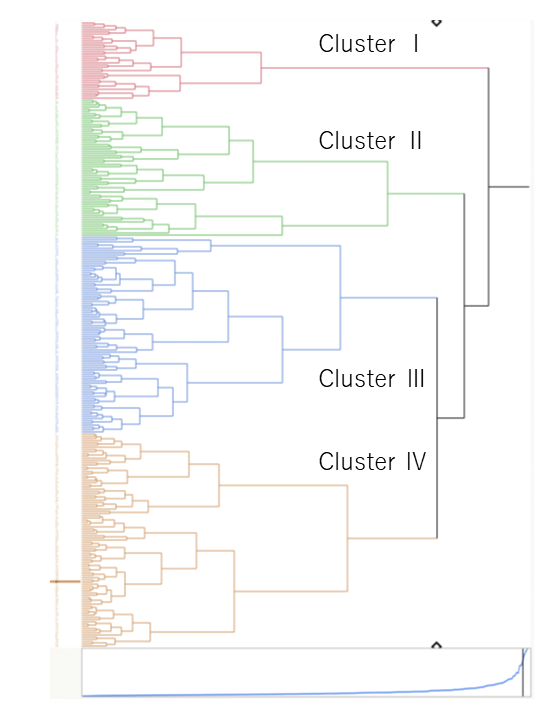


Four clusters were identified in the cluster analysis using clinical data.

IIP, idiopathic interstitial pneumonia

**Supplementary Figure 2. Overall survival in the new clusters derived from Cluster II**


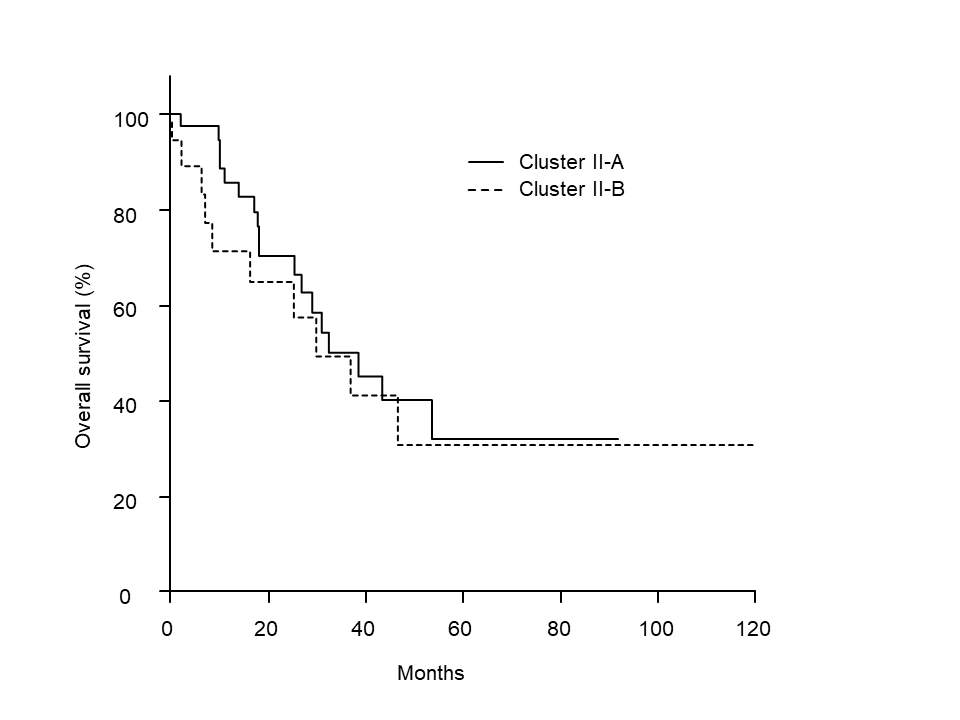


Overall survival did not differ between the new two clusters.

**Supplementary Figure 3.** **Cumulative incidence of acute exacerbations in the new clusters derived from Cluster II**


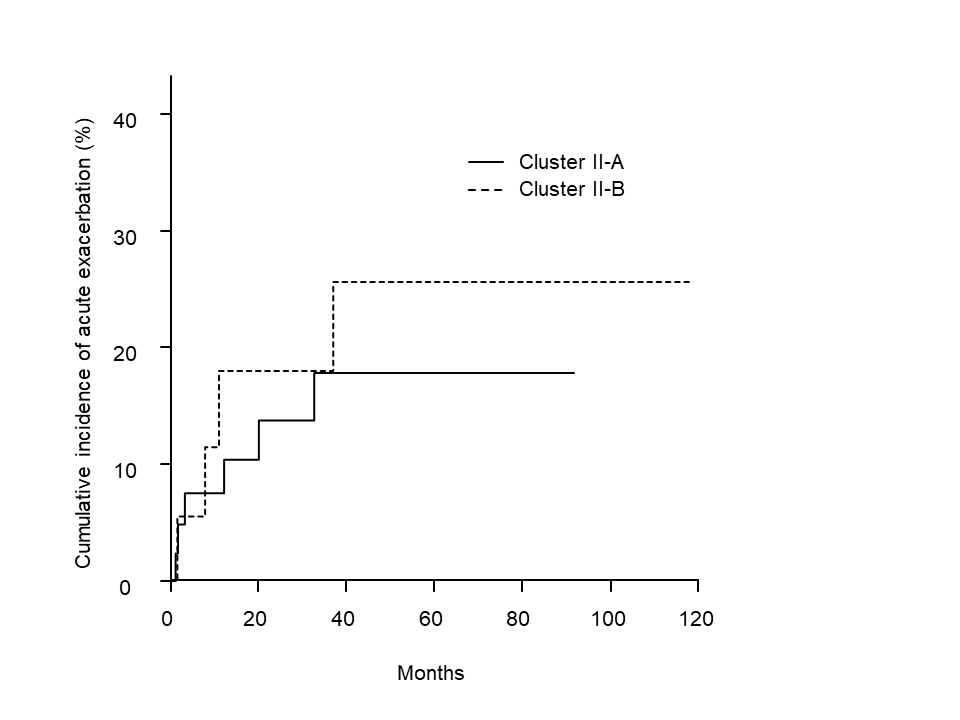


The cumulative incidence of acute exacerbations did not differ between the new two clusters.
