# Supplementary material for: Cluster analysis-based clinical phenotypes of idiopathic interstitial pneumonias: associations with acute exacerbation and overall survival
Source: BMC Pulm Med. 2021 Feb 22;21:63. doi: 10.1186/s12890-021-01428-3 (PMC7898746; doi:10.1186/s12890-021-01428-3)
Supplement: Supplementary file 2 — Additional file 2. Supplementary Tables 1–4: Table 1. Cox proportional hazard analysis of overall survival. Table 2. Cox proportional hazard analysis of acute exacerbations. Table 3. Characteristics of the new clusters derived from Cluster II. Table 4. Diagnosis of IIPs in the new clusters derived from Cluster II. [file 12890_2021_1428_MOESM2_ESM.docx]

**Cluster analysis-based clinical phenotypes of idiopathic interstitial pneumonias: Associations with acute exacerbation and overall survival**

**Authors:** Yoichiro Aoshima, MD^1^, Masato Karayama, MD, PhD^1^, Yasuoki Horiike, MD^1^, Kazutaka Mori, MD, PhD^2^, Hideki Yasui, MD, PhD^1^, Hironao Hozumi, MD, PhD^1^, Yuzo Suzuki, MD, PhD^1^, Kazuki Furuhashi, MD, PhD^1^, Tomoyuki Fujisawa, MD, PhD^1^, Noriyuki Enomoto, MD, PhD^1^, Yutaro Nakamura, MD, PhD^1^, Naoki Inui, MD, PhD^1,3^, Takafumi Suda, MD, PhD^1^

**Affiliations:**

^1^Second Division, Department of Internal Medicine, Hamamatsu University School of Medicine, 1-20-1 Handayama, Hamamatsu 431-3192, Japan

^2^Department of Respiratory Medicine, Shizuoka City Shimizu Hospital, 1231 Miyakami, Shizuoka, 424-8636, Japan

^3^Department of Clinical Pharmacology and Therapeutics, Hamamatsu University School of Medicine, 1-20-1 Handayama, Hamamatsu 431-3192, Japan

**Corresponding author:** Masato Karayama MD, PhD

**Supplementary Table 1. Cox proportional hazard analysis of overall survival**

|  | Univariate analysis | | | Multivariate analysis | | |
| --- | --- | --- | --- | --- | --- | --- |
|  | HR | 95% CI | *p*-values | HR | 95% CI | *p*-values |
| Age* | 1.06 | 1.03 - 1.09 | <0.01 | 1.04 | 1.01- 1.07 | <0.01 |
| Sex, male | 1.66 | 0.91 - 3.03 | 0.09 |  |  |  |
| Body mass index* | 0.98 | 0.91 - 1.05 | 0.63 |  |  |  |
| Pack-year smoking* | 0.99 | 0.98 - 1.00 | 0.12 |  |  |  |
| Dust exposure | 1.16 | 0.69 - 1.96 | 0.56 |  |  |  |
| Autoimmune features | 1.22 | 0.64 - 2.32 | 0.53 |  |  |  |
| Spirometry |  |  |  |  |  |  |
| % predicted FVC* | 0.96 | 0.95 - 0.98 | <0.01 | 0.97 | 0.95 – 0.99 | 0.02 |
| % predicted FEV₁* | 0.96 | 0.95 - 0.98 | <0.01 | 0.98 | 0.96 – 1.00 | 0.25 |
| Laboratory data |  |  |  |  |  |  |
| CRP (mg/dL)* | 1.02 | 0.96 - 1.08 | 0.45 |  |  |  |
| LDH (U/L)** | 1.02 | 0.99 - 1.05 | 0.14 |  |  |  |
| Albumin (g/dL)* | 0.46 | 0.31 - 0.70 | <0.01 | 0.58 | 0.35 – 0.94 | 0.02 |
| KL-6 (U/mL)*** | 1.01 | 0.99 - 1.03 | 0.12 |  |  |  |
| SP-D (ng/mL)*** | 1.12 | 0.99 – 1.25 | 0.05 |  |  |  |
| CT findings |  |  |  |  |  |  |
| Emphysema | 1.43 | 0.90 - 2.28 | 0.12 |  |  |  |
| Honeycombing | 2.24 | 1.40 - 3.57 | <0.01 | 1.88 | 1.16 – 3.04 | 0.02 |

CI, confidence interval; CRP, C-reactive protein; FEV_1_, forced expiratory volume in 1 s; FVC, forced vital capacity; HR, hazard ratio; KL-6, Krebs von den Lungen-6; LDH, lactate dehydrogenase; SP-D, pulmonary surfactant protein-D

*Hazard ratio was expressed as per 1 increase

**Hazard ratio was expressed as per 10 increase

**Hazard ratio was expressed as per 100 increase

**Supplementary Table 2. Cox proportional hazard analysis of acute exacerbations**

|  | Univariate analysis | | | Multivariate analysis | | |
| --- | --- | --- | --- | --- | --- | --- |
|  | HR | 95% CI | *p*-values | HR | 95% CI | *p*-values |
| Age* | 1.01 | 0.97 - 1.05 | 0.51 |  |  |  |
| Sex, male | 0.76 | 0.37 - 1.55 | 0.45 |  |  |  |
| Body mass index* | 1.03 | 0.93 - 1.14 | 0.51 |  |  |  |
| Pack-year smoking* | 0.99 | 0.98 - 1.01 | 0.56 |  |  |  |
| Dust exposure | 1.65 | 0.83 - 3.29 | 0.15 |  |  |  |
| Autoimmune features | 1.12 | 0.43 - 2.88 | 0.81 |  |  |  |
| Spirometry |  |  |  |  |  |  |
| % predicted FVC* | 0.96 | 0.94 - 0.97 | <0.01 | 0.95 | 0.92 – 0.97 | <0.01 |
| % predicted FEV₁* | 0.96 | 0.94 - 0.98 | <0.01 | 1.00 | 0.97 – 1.03 | 0.65 |
| Laboratory data |  |  |  |  |  |  |
| CRP (mg/dL)* | 1.02 | 0.94 - 1.11 | 0.50 |  |  |  |
| LDH (U/L)** | 1.04 | 1.00 - 1.08 | 0.02 | 1.06 | 1.02 – 1.11 | 0.05 |
| Albumin (g/dL)* | 0.55 | 0.28 - 1.05 | 0.07 |  |  |  |
| KL-6 (U/mL)*** | 1.03 | 1.00 - 1.05 | 0.01 | 1.03 | 0.99 – 1.06 | 0.06 |
| SP-D (ng/mL)*** | 1.13 | 0.97 – 1.32 | 0.10 |  |  |  |
| CT findings |  |  |  |  |  |  |
| Emphysema | 1.09 | 0.54 - 2.19 | 0.79 |  |  |  |
| Honeycombing | 3.23 | 1.67 - 6.25 | <0.01 | 3.86 | 1.92 – 7.73 | <0.01 |

CI, confidence interval; CRP, C-reactive protein; FEV_1_, forced expiratory volume in 1 s; FVC, forced vital capacity; HR, hazard ratio; KL-6, Krebs von den Lungen-6; LDH, lactate dehydrogenase; SP-D, pulmonary surfactant protein-D

*Hazard ratio was expressed as per 1 increase

**Hazard ratio was expressed as per 10 increase

**Hazard ratio was expressed as per 100 increase

**Supplementary Table 3. Characteristics of the new clusters derived from Cluster II**

|  | Cluster II-A, n=43 | Cluster II-B, n=19 |
| --- | --- | --- |
| Age | 74.1 (60-90) | 76.7 (55-85) |
| Sex, male | 31 (72.0)* | 18 (94.7) |
| Body mass index | 19.4 (13.1-24.6)* | 22.5 (19.1-25.0) |
| Smoking history | 18 (41.8) | 13 (68.4) |
| Pack-year smoking | 28 (5-70.5) | 42 (5-100) |
| Dust exposure  Organic materials  Inorganic materials | 2 (4.6)  0 (0)  2 (4.6) | 4 (21.0)  0 (0)  4 (21.0) |
| Autoimmune features | 6 (13.9) | 0 (0) |
| Spirometry |  |  |
| % predicted FVC | 57.6 (27.6-81.5)* | 77.4 (116.0-46.6) |
| % predicted FEV₁ | 65.5 (31.9-100.0)* | 85.7 (51.6-94.0) |
| FEV₁/FVC | 90.6 (63.4-100.0)* | 80.4 (67.1-98.8) |
| Laboratory data |  |  |
| CRP (mg/dL) | 1.39 (0.02-16.9)* | 6.98 (1.14-25.5) |
| LDH (U/L) | 212 (132-315)* | 245 (179-450) |
| Albumin (g/dL) | 3.8 (2.5-4.5)* | 3.1 (1.9-3.9) |
| KL-6 (U/mL) | 786 (112-2070) | 590 (122-2063) |
| SP-D (ng/mL) | 189 (33.4-1130) | 149 (46.4-360) |
| CT findings |  |  |
| Emphysema | 8 (18.6) | 6 (31.5) |
| Honeycombing | 17 (39.5) | 6 (31.5) |
| Treatment |  |  |
| Steroids | 11 (25.5)* | 10 (52.6) |
| Immunosuppressants | 4 (9.3) | 1 (5.2) |
| Antifibrotic agents | 12 (27.9)* | 1 (5.2) |

Data are presented as median (range) or number (%).

**p* < 0.05 compared with Cluster II-B

**Supplementary Table 4. Diagnosis of IIPs in the new clusters derived from Cluster II**

|  | Cluster II-A, n=43 | Cluster II-B, n=19 |
| --- | --- | --- |
| IPF | 14 (32.5) | 8 (42.1) |
| NSIP | 0 (0) | 0 (0) |
| COP | 3 (6.9) | 5 (26.3) |
| DIP / RB-ILD | 0 (0) | 0 (0) |
| PPFE | 8 (18.6) | 0 (0) |
| Unclassifiable IIPs | 18 (41.8) | 6 (31.5) |

Data are expressed as number (%).

COP, cryptogenic organizing pneumonia; DIP, desquamative interstitial pneumonia; IIP, idiopathic interstitial pneumonia; IPAF, interstitial pneumonia with autoimmune features; IPF, idiopathic pulmonary fibrosis; NSIP, non-specific interstitial pneumonia; PPFE, pleuroparenchymal fibroelastosis; RB-ILD, respiratory bronchiolitis-associated interstitial lung disease
